# Supplementary figures and images for: A dual role for SAMHD1 in regulating HBV cccDNA and RT-dependent particle genesis
Source: Life Sci Alliance. 2019 Mar 27;2(2):e201900355. doi: 10.26508/lsa.201900355 (PMC6438393; doi:10.26508/lsa.201900355)

Fig 1A

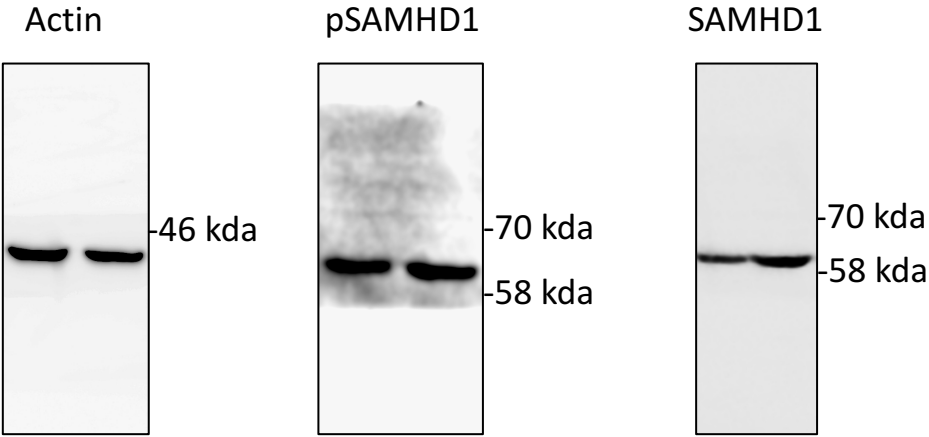

Fig 1B

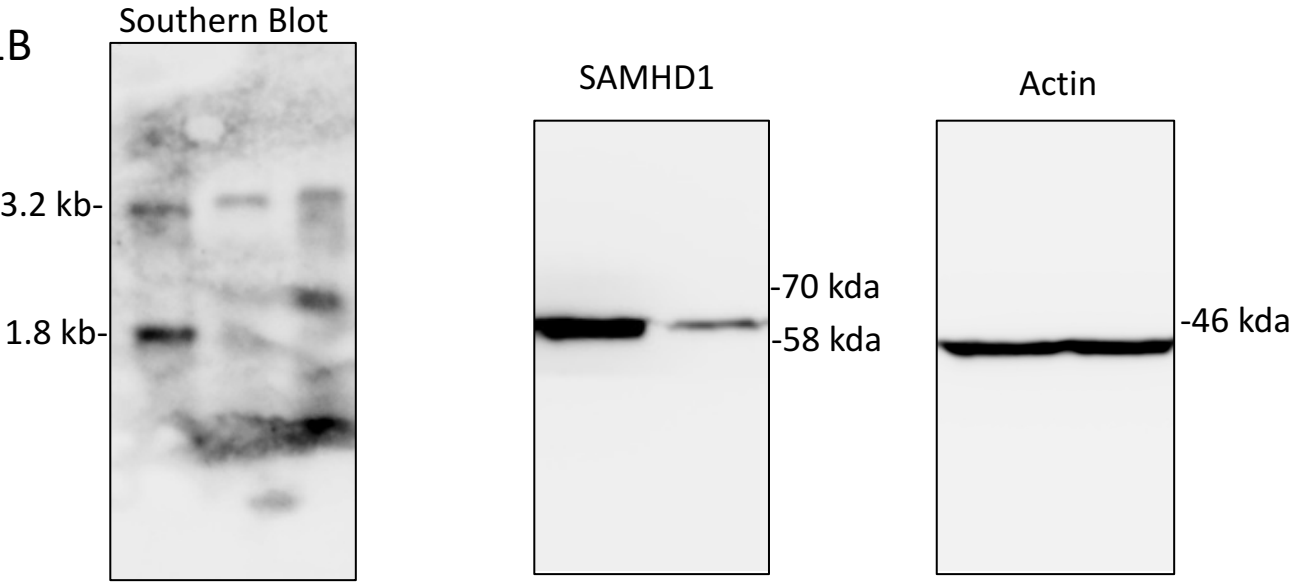

Fig 1C

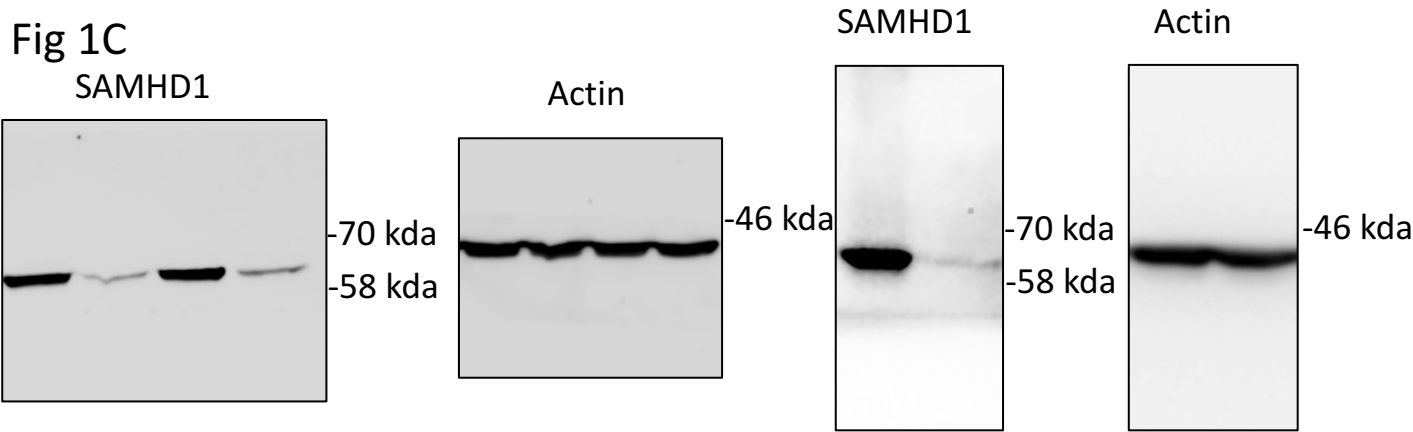

Supplement: Supplementary file 1 [file LSA-2019-00355_SdataF1.pdf]

Fig 2a

SAMHD1

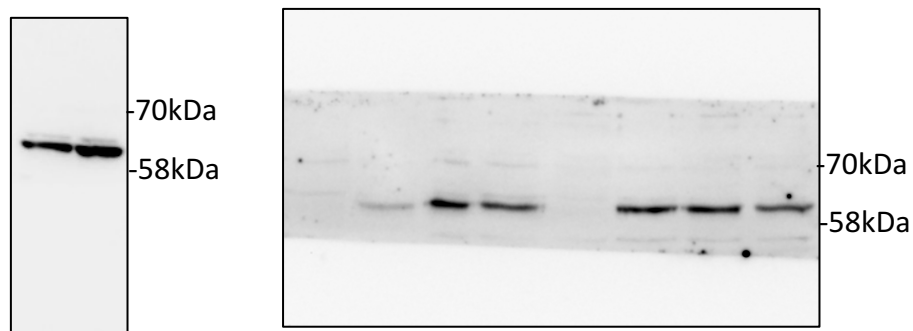

Actin

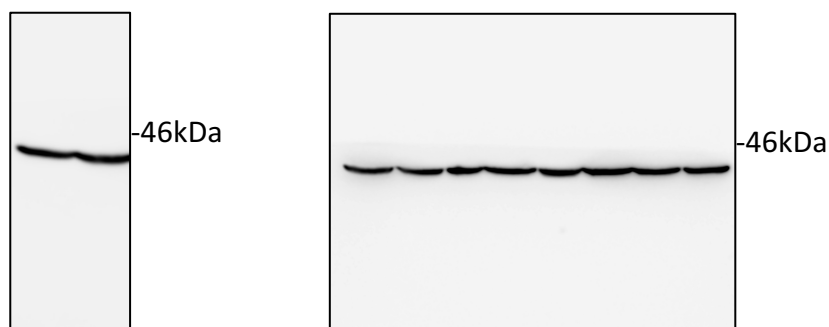

Fig 2e

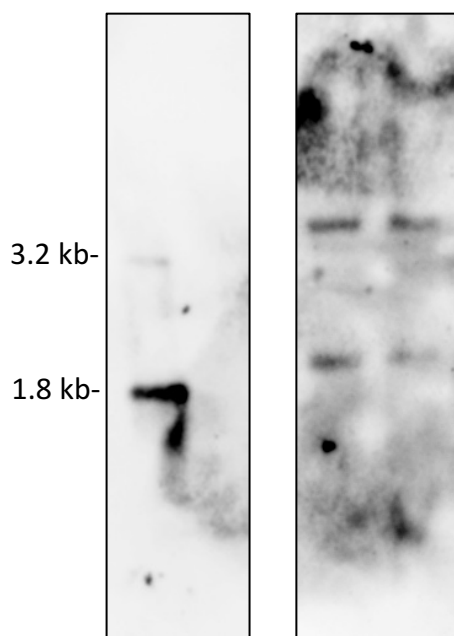

Supplement: Supplementary file 2 [file LSA-2019-00355_SdataF2.pdf]

Fig 3b

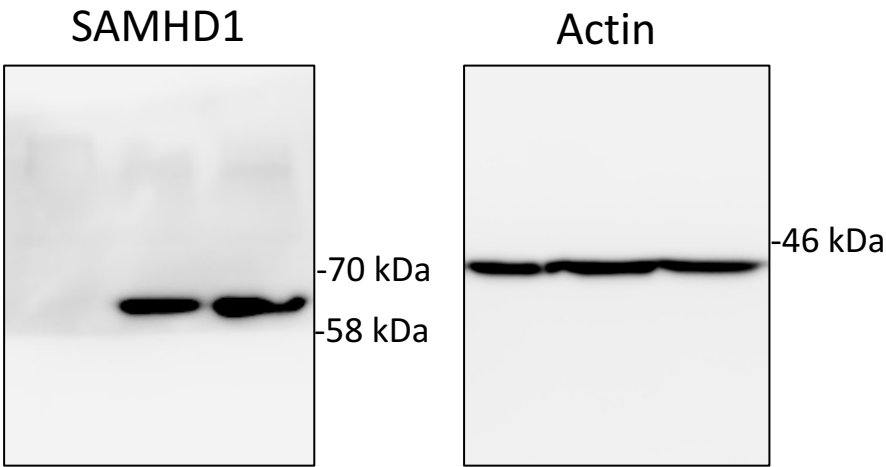

Fig 3c

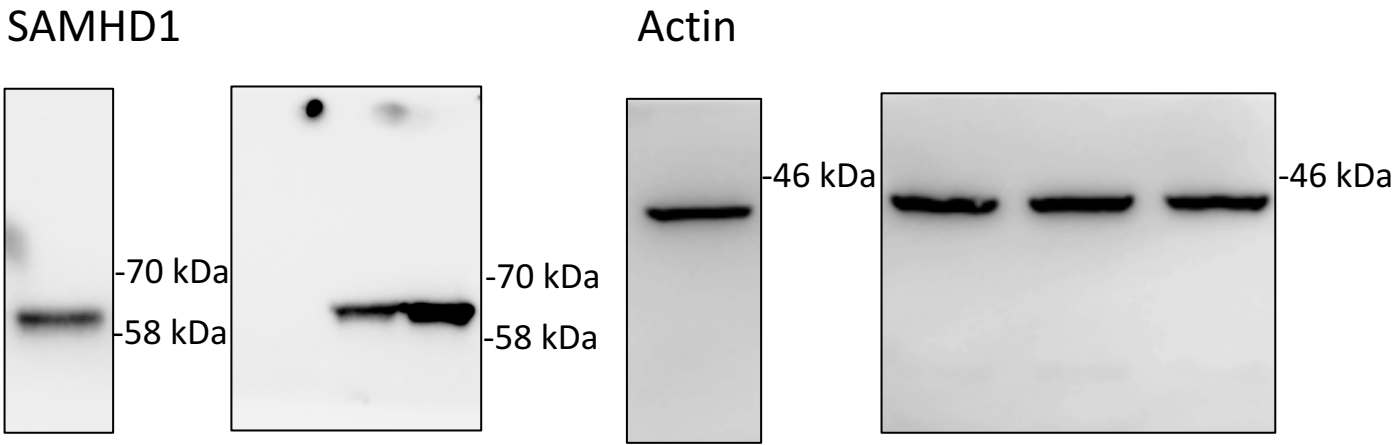

Supplement: Supplementary file 3 [file LSA-2019-00355_SdataF3.pdf]

Fig 5A

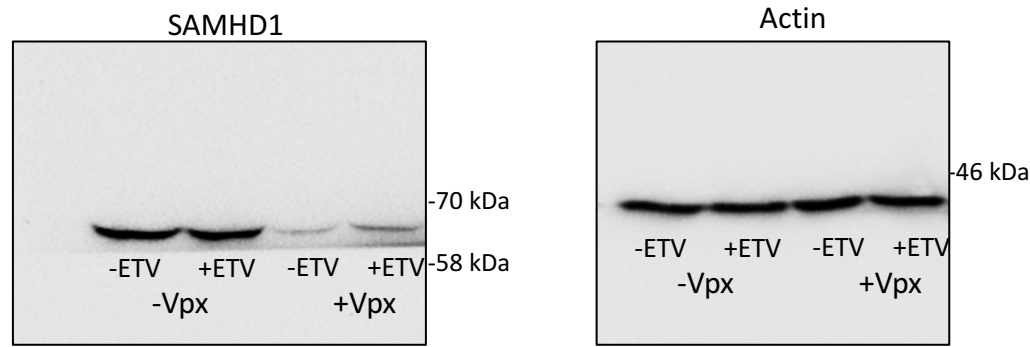

Fig 5B

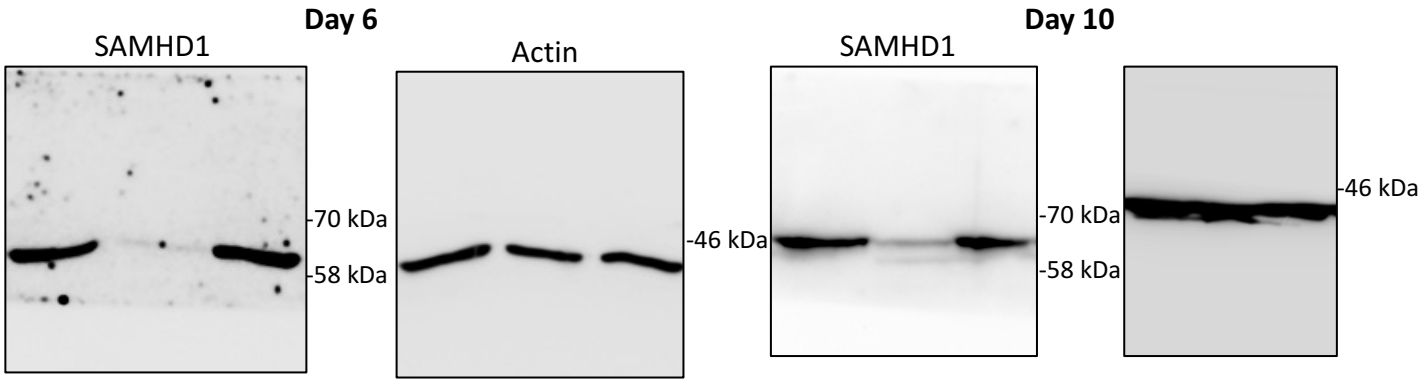

Fig 5C

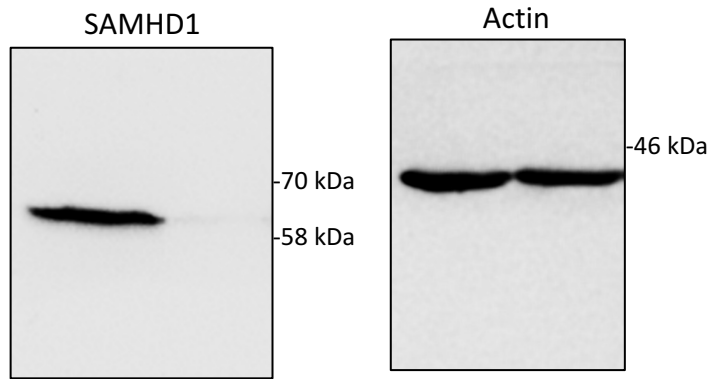

Supplement: Supplementary file 4 [file LSA-2019-00355_SdataF5.pdf]
